# 506. Resistance Analyses from the Remdesivir Phase 3 REDPINE Study in Participants with Severely Reduced Kidney Function Who Were Hospitalized for COVID-19

**DOI:** 10.1093/ofid/ofad500.575

**Published:** 2023-11-27

**Authors:** Kristen Andreatta, Jiani Li, Lauren Rodriguez, Dong Han, Simin Xu, Jason K Perry, Yiannis Koullias, Robert H Hyland, Charlotte Hedskog

**Affiliations:** Gilead Sciences, Inc., Foster City, California; Gilead Sciences, Inc., Foster City, California; Gilead Sciences, Inc., Foster City, California; Gilead Sciences, Inc., Foster City, California; Gilead Sciences, Inc., Foster City, California; Gilead Sciences, Inc., Foster City, California; Gilead Sciences, Inc., Foster City, California; Gilead Sciences, Inc., Foster City, California; Gilead Sciences, Inc., Foster City, California

## Abstract

**Background:**

Remdesivir (RDV) is a broad-spectrum nucleotide analog prodrug approved for the treatment of COVID-19 in nonhospitalized and hospitalized adult and pediatric patients with clinical benefit demonstrated in multiple Phase 3 trials. Here we present SARS-CoV-2 resistance analyses from the Phase 3 REDPINE study, which demonstrated the safety of RDV in participants with severely reduced kidney function.

**Methods:**

REDPINE (NCT04745351) was a double-blind, placebo-controlled trial in participants hospitalized for COVID-19 with severely reduced kidney function who were randomized 2:1 to receive RDV or placebo for 5 days. Full-genome deep sequencing of SARS-CoV-2 was performed on nasopharyngeal swab samples collected on Days 1 (baseline), 3, 5, 7, 14, 21, and/or 29. Emergent amino acid substitutions from RDV-treated participants were tested for RDV susceptibility in a SARS-CoV-2 replicon system.

**Results:**

Of the 243 participants randomized and treated, 82 (RDV, 55; placebo, 27) met the resistance analysis criteria (all participants with all-cause death or invasive mechanical ventilation [IMV] through Day 29 or with SARS-CoV-2 RNA above the viral load assay lower limit of quantitation on Day 14). Sequencing data at both baseline and postbaseline timepoints were obtained from 60 participants (RDV, 41; placebo, 19). Among these, emergent amino acid substitutions in Nsp12 were observed in 8/41 (19.5%) in the RDV group and 1/19 (5.3%) in the placebo group. The proportions of participants with all-cause death or IMV through Day 29 were comparable between participants in the RDV group with emergent Nsp12 substitutions (5/8, 62.5%) or without emergent Nsp12 substitutions (30/33, 90.9%). In 4 participants in the RDV group, Nsp12 substitutions with low-level reduced susceptibility to RDV were identified in samples collected 9 days after cessation of RDV treatment (2.9- to 3.4-fold change in EC_50_): M794I (n = 2), C799F (n = 1), and E136V (n = 1). Three of these 4 participants had received solid organ transplants and were receiving concomitant immunosuppressive therapies during the study.

**Conclusion:**

The results of resistance analyses in participants with severely reduced kidney function hospitalized for COVID-19 confirm a high barrier to clinically meaningful resistance to RDV in COVID-19 patients.

**Disclosures:**

**Kristen Andreatta, MS**, Gilead Sciences, Inc.: Employee|Gilead Sciences, Inc.: Stocks/Bonds **Jiani Li, PhD**, Gilead Sciences, Inc.: Employee|Gilead Sciences, Inc.: Stocks/Bonds **Lauren Rodriguez, PhD**, Gilead Sciences, Inc.: Employee|Gilead Sciences, Inc.: Stocks/Bonds **Dong Han, MS**, Gilead Sciences, Inc.: Employee|Gilead Sciences, Inc.: Stocks/Bonds **Simin Xu, MS**, Gilead Sciences, Inc.: Employee|Gilead Sciences, Inc.: Stocks/Bonds **Jason K. Perry, PhD**, Gilead Sciences, Inc.: Employee|Gilead Sciences, Inc.: Stocks/Bonds **Yiannis Koullias, MD**, Gilead Sciences, Inc.: Employee|Gilead Sciences, Inc.: Stocks/Bonds **Robert H. Hyland, DPhil**, Gilead Sciences, Inc.: Employee|Gilead Sciences, Inc.: Stocks/Bonds **Charlotte Hedskog, PhD**, Gilead Sciences, Inc.: Employee|Gilead Sciences, Inc.: Stocks/Bonds

